# Supplementary figures and images for: Creating clear and informative image-based figures for scientific publications
Source: PLoS Biol. 2021 Mar 31;19(3):e3001161. doi: 10.1371/journal.pbio.3001161 (PMC8041175; doi:10.1371/journal.pbio.3001161)

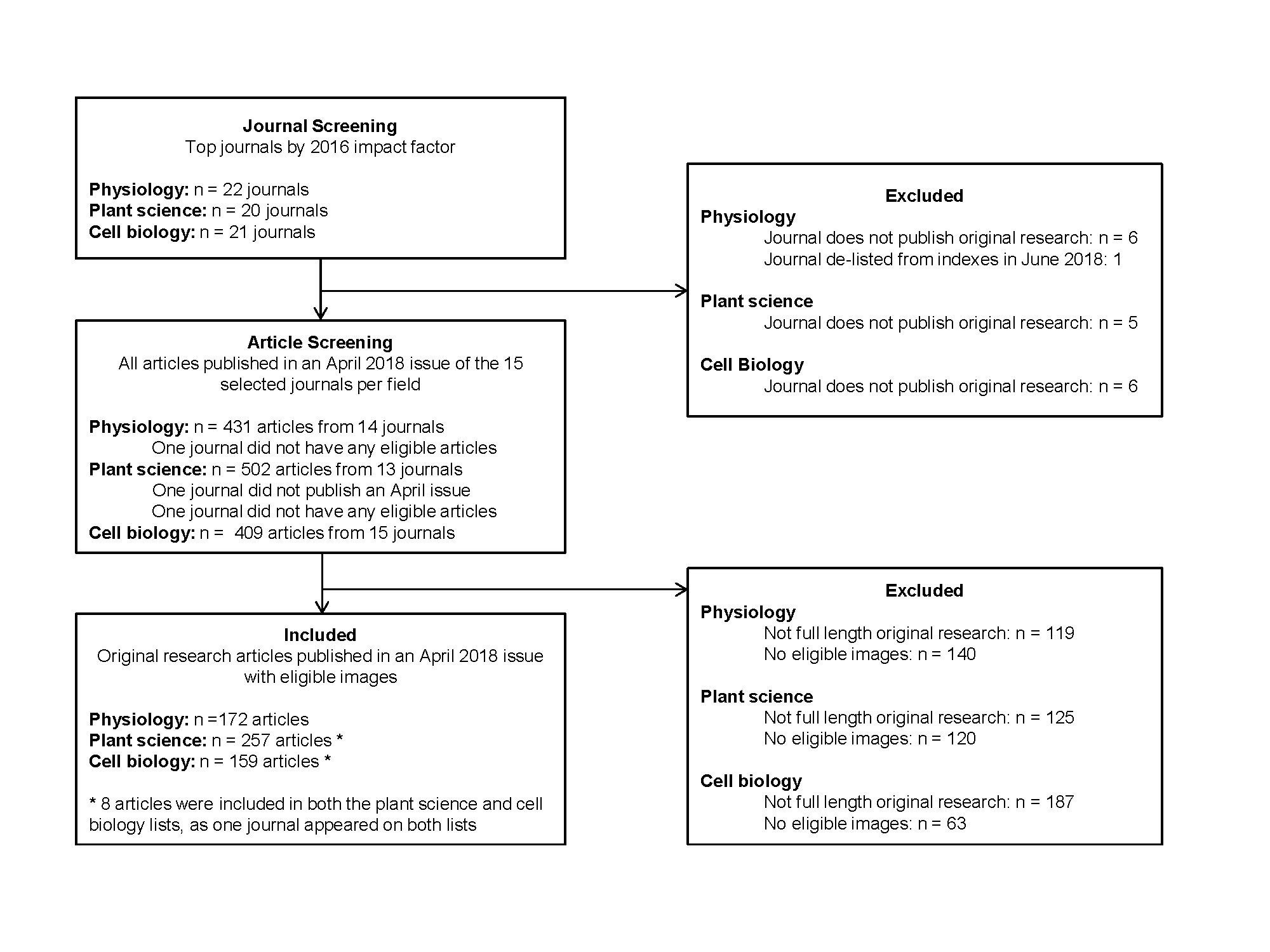

Supplement: S1 Fig — This flow chart illustrates the number of included and excluded journals or articles, along with reasons for exclusion, at each stage of the study. (JPG) [file pbio.3001161.s001.jpg]
