# Supplementary material for: Creating clear and informative image-based figures for scientific publications
Source: PLoS Biol. 2021 Mar 31;19(3):e3001161. doi: 10.1371/journal.pbio.3001161 (PMC8041175; doi:10.1371/journal.pbio.3001161)
Supplement: S1 Table — Values are n, or n (% of all articles). Screening was performed to exclude articles that were not full-length original research articles (e.g., reviews, editorials, perspectives, commentaries, letters to the editor, short communications, etc.), were not published in April 2018, or did not include eligible images. AJP, American Journal of Physiology. (DOCX) [file pbio.3001161.s002.docx]

| **S1 Table:** Number of articles examined by journal in physiology | | | | |
| --- | --- | --- | --- | --- |
| **Journal** | **Articles Screened**  (n = 431) | **Original Research Articles** (n = 312, 72%) | | **Included Articles**  (n = 172, 40%) |
| Journal of Pineal Research | 7 | 6 (86%) | 5 (71%) | |
| Acta Physiologica | 21 | 10 (48%) | 5 (24%) | |
| Journal of Physiology | 39 | 22 (56%) | 12 (31%) | |
| International Journal of Behavioral Nutrition and Physical Activity | 9 | 9 (100%) | 0 | |
| AJP: Lung, Cellular and Molecular Physiology | 15 | 12 (80%) | 6 (40%) | |
| Journal of General Physiology | 10 | 4 (40%) | 3 (30%) | |
| AJP: Endocrinology and Metabolism | 9 | 8 (89%) | 6 (67%) | |
| Frontiers in Physiology | 142 | 107 (75%) | 47 (33%) | |
| Journal of Cellular Physiology | 88 | 55 (63%) | 47 (53%) | |
| AJP: Renal Physiology | 15 | 15 (100%) | 10 (67%) | |
| AJP: Cell Physiology | 11 | 11 (100%) | 9 (82%) | |
| Journal of Biological Rhythms | 9 | 8 (89%) | 2 (22%) | |
| AJP: Gastrointestinal and Liver Physiology | 6 | 6 (100%) | 5 (83%) | |
| Journal of Applied Physiology | 31 | 31 (100%) | 10 (32%) | |
| AJP: Heart and Circulatory Physiology | 19 | 8 (42%) | 5 (26%) | |
| Values are n, or n (% of all articles). Screening was performed to exclude articles that were not full-length original research articles (e.g. reviews, editorials, perspectives, commentaries, letters to the editor, short communications, etc.), were not published in April 2018, or did not include eligible images.  Abbreviations: AJP, American Journal of Physiology | | | | |
