# Supplementary material for: Creating clear and informative image-based figures for scientific publications
Source: PLoS Biol. 2021 Mar 31;19(3):e3001161. doi: 10.1371/journal.pbio.3001161 (PMC8041175; doi:10.1371/journal.pbio.3001161)
Supplement: S2 Table — Values are n, or n (% of all articles). Screening was performed to exclude articles that were not full-length original research articles (e.g., reviews, editorials, perspectives, commentaries, letters to the editor, short communications, etc.), were not published in April 2018, or did not include eligible images. *This journal was also included on the cell biology list (Table S3). **No articles from the Journal of Ecology were screened as the journal did not publish an April 2018 issue. (DOCX) [file pbio.3001161.s003.docx]

| **S2 Table:** Number of articles examined by journal in plant science | | | |
| --- | --- | --- | --- |
| **Journal** | **Articles Screened**  (n = 502) | **Original Research Articles**  (n = 377, 75%) | **Included Articles**  (n = 257, 51%) |
| Nature Plants | 13 | 3 (23%) | 0 |
| Molecular Plant | 14 | 7 (50%) | 6 (43%) |
| Plant Cell * | 15 | 9 (60%) | 8 (53%) |
| Plant Biotechnology Journal | 12 | 10 (83%) | 6 (50%) |
| New Phytologist | 73 | 53 (73%) | 31 (42%) |
| Plant Physiology | 39 | 34 (87%) | 27 (69%) |
| Plant Cell and Environment | 14 | 11 (79%) | 7 (50%) |
| Plant Journal | 31 | 24 (77%) | 19 (61%) |
| Journal of Experimental Botany | 74 | 55 (74%) | 41 (55%) |
| Journal of Ecology ** | 0 |  |  |
| Plant and Cell Physiology | 21 | 13 (62%) | 9 (43%) |
| Molecular Plant Pathology | 21 | 16 (76%) | 15 (71%) |
| Environmental and Experimental Botany | 17 | 17 (100%) | 12 (71%) |
| Molecular Plant – Microbiome Interactions | 8 | 7 (88%) | 4 (50%) |
| Frontiers in Plant Science | 150 | 118 (79%) | 72 (48%) |
| * This journal was also included on the cell biology list (S3 Table).  ** No articles from the Journal of Ecology were screened as the journal did not publish an April 2018 issue.  Values are n, or n (% of all articles). Screening was performed to exclude articles that were not full-length original research articles (e.g. reviews, editorials, perspectives, commentaries, letters to the editor, short communications, etc.), were not published in April 2018, or did not include eligible images. | | | |
