# Supplementary material for: Creating clear and informative image-based figures for scientific publications
Source: PLoS Biol. 2021 Mar 31;19(3):e3001161. doi: 10.1371/journal.pbio.3001161 (PMC8041175; doi:10.1371/journal.pbio.3001161)
Supplement: S3 Table — Values are n, or n (% of all articles). Screening was performed to exclude articles that were not full-length original research articles (e.g., reviews, editorials, perspectives, commentaries, letters to the editor, short communications, etc.), were not published in April 2018, or did not include eligible images. *This journal was also included on the plant science list (Table S2). (DOCX) [file pbio.3001161.s004.docx]

| **S3 Table:** Number of articles examined by journal in cell biology | | | |
| --- | --- | --- | --- |
| **Journal** | **Articles Screened**  (n = 409) | **Original Research Articles**  (n = 222, 54%) | **Included Articles**  (n = 159, 39%) |
| Cell | 50 | 33 (76%) | 19 (38%) |
| Nature medicine | 32 | 10 (31%) | 6 (19%) |
| Cancer Cell | 21 | 12 (57%) | 5 (24%) |
| Cell Stem Cell | 18 | 7 (39%) | 5 (28%) |
| Nature Cell Biology | 20 | 9 (45%) | 9 (45%) |
| Cell Metabolism | 20 | 9 (45%) | 8 (40%) |
| Science Translational Medicine | 25 | 18 (72%) | 17 (68%) |
| Cell Research | 13 | 6 (46%) | 5 (38%) |
| Molecular Cell | 38 | 26 (68%) | 13 (34%) |
| Nature Structural and Molecular Biology | 12 | 7 (58%) | 2 (17%) |
| EMBO Journal | 23 | 17 (74%) | 16 (70%) |
| Genes and Development | 13 | 8 (62%) | 5 (38%) |
| Developmental Cell | 22 | 15 (68%) | 15 (68%) |
| Current Biology | 87 | 36 (41%) | 26 (30%) |
| Plant Cell * | 15 | 9 (60%) | 8 (53%) |
| * This journal was also included on the plant science list (S2 Table).  Values are n, or n (% of all articles). Screening was performed to exclude articles that were not full length original research articles (e.g. reviews, editorials, perspectives, commentaries, letters to the editor, short communications, etc.), were not published in April 2018, or did not include eligible images. | | | |
