# Supplementary material for: Creating clear and informative image-based figures for scientific publications
Source: PLoS Biol. 2021 Mar 31;19(3):e3001161. doi: 10.1371/journal.pbio.3001161 (PMC8041175; doi:10.1371/journal.pbio.3001161)
Supplement: S4 Table — Values are percent of papers. (DOCX) [file pbio.3001161.s005.docx]

| **S4 Table:** Scale information in papers | | | | | | | |
| --- | --- | --- | --- | --- | --- | --- | --- |
| **Field** | **No scale information in any figure** | **Some scale information** | | | | **Complete scale information** | |
|  |  | Some figures, magnification in legend | All figures, magnification in legend | Some figures, scale bar with dimensions in legend | Some figures, scale bar with dimensions | All figures, scale bar with dimensions in legend | All figures, scale bar with dimensions |
| Physiology | 24.4 | 5.2 | 1.7 | 10.5 | 9.3 | 26.7 | 22.1 |
| Cell biology | 10.1 | 0.0 | 1.3 | 22.0 | 11.9 | 40.9 | 13.8 |
| Plant science | 29.2 | 0.4 | 0.4 | 31.5 | 10.5 | 23.3 | 4.7 |
| Values are % of papers. | | | | | | | |
