# Supplementary material for: Creating clear and informative image-based figures for scientific publications
Source: PLoS Biol. 2021 Mar 31;19(3):e3001161. doi: 10.1371/journal.pbio.3001161 (PMC8041175; doi:10.1371/journal.pbio.3001161)
Supplement: Attachment — Submitted filename: Response_to_reviewers_R1_20200126.docx [file pbio.3001161.s006.docx]

**Key Revisions**

We thank the reviewers for their detailed comments and suggestions to improve the paper. Here we respond to a few comments raised by multiple reviewers. A point-by-point response to reviewers follows this summary.

***Some reviewers requested examples of problematic images taken from the papers included in our dataset.*** While the authors understand the desire for “real world” examples, highlighting flaws in published papers written by others is a risky strategy. In addition to potential copyright issues, this also introduces the potential for backlash from authors whose work is highlighted in a negative way. We therefore chose to create our own examples, based on problems observed by the data abstractors, to ensure that the paper is solution oriented. This also allows us to use the same images to illustrate poor vs. improved practices, making it easier for authors to compare and contrast. The types of problems shown were selected based on feedback from the data abstractors, who systematically examined every (non-blot) image in hundreds of papers. We have clarified that we have presented simulated examples, based on practices observed during data abstraction, in the methods section and in selected figure legends.

***Several reviewers asked about the error rates:*** The methodology for these calculations was addressed in the “Verification” section of the methods. We have expanded this section to answer reviewer questions and reported the calculated error rates, so that they do not show up without warning in the limitations section.

***Several reviewers observed that we did not follow all recommended practices in all figures:*** As noted in the manuscript, illustrating every practice described in every figure of the paper would lead to overplotting and make it difficult to identify the key concept shown in a particular figure. A central tenet of designing good visualizations is that information that is not essential to the message of the figure should be removed to avoid distraction. This creates some conceptual challenges. For example, species and tissue information is not essential to the message of the paper because we are using images to illustrate poor vs. improved practices in general, rather than to show scientific content related to the image itself. We have chosen to model good practice for this criteria in every figure, as it affects the legend and does not add clutter to the figure itself.

**Key revisions:** We added scale bars to all image-based figures, except figure 5 (a note appears in the legend explaining this). In cases where we have chosen to use poor practices highlighted in other figures to demonstrate concepts, these choices are now explained in the figure legends.

***Referring to panels:*** To increase clarity, we have numbered the panels in all figures and now refer to Fig 1A panel 1, 2, 3, etc.

 Reviewer 1, Elisabeth Bik

In this paper, the authors screened hundreds of papers from three different scientific fields (physiology, cell biology, and plant sciences) and selected 580 papers that included photographic images. They analyzed the papers containing photographic images for the presence of scale bars, inset annotation, clear labeling, colorblindness-friendly color scheme, adequate description of the specimen etc. The majority of the papers failed one of these criteria. Examples of good and bad image labeling are given throughout the manuscript.

The paper is a welcome addition to the field of meta-science (science about science papers, and provides clear guidelines about what constitutes good labeling and color-use of photographic images in biomedical papers. The search strategy is clearly described and reproducible, and the paper was easy to read and understand. Also, kudos to the authors for including an image featuring Darth Vader.

**I have some minor comments.**

**General comments:**

The term "Microphotograph" might benefit from a definition. It appears the authors mean a photo taken from a specimen under a microscope (e.g. of cells or tissues), but I am not sure. Is a "Photograph" then defined as a photo of something visible to the eye such as a plant or a petridish? One could call all the image types mentioned in Figure 1A "photographs", so maybe consider using the term "macrophotograph" for a photo that is not a microphotograph.

**Authors:** Thank you for helping to clarify the manuscript. We have changed “microphotograph” to “microscopy image” throughout the paper to clarify that this category refers to images captured through a microscope.

**R1:** Are the examples shown in Figure 4-6 from the papers that were screened for this paper? Or were they taken from public sources (as indicated for some photos) and then manipulated digitally to either remove or add a scale bar (see fig 4)? It would be nice to clearly define that in the Methods (or maybe I missed that).

**Authors:** We have added a sentence to the legends of figure 3, 4, 12 and 13 noting that examples of problematic images were created based on problems observed by the reviewers.

**Specific comments**

**R1:** Results. Page 4. "More than half of the papers in the sample contained images (plant science: 68%, cell biology: 72%, physiology: 55%)." - These numbers do not seem to match the data provided in Supplemental Tables 1-3. Maybe I am misunderstanding something, but Supplemental Tables 1-3 mention 39.9, 51.2, and 38.9% of papers, which are much lower numbers.

Physiology: 431 screened - 172 included (39.9%)

Plant science: 502 screened - 257 included (51.2%)

Cell Biology: 409 screened - 159 included (38.9%)

**Authors:** The supplemental tables examine papers that were excluded because they were not original research, as well as original research articles excluded because they did not contain images. The data presented in the paper describe the number of original research articles that contain images. We have adjusted the text in the supplemental tables and results to clarify this and added an additional column showing original research articles to the supplemental tables; this information is more clearly described in the study flow chart (figure S1). The supplemental tables are mainly for readers who are intimately familiar with a field and want to see information for individual journals.

**R1:** Page 22: What is meant by "Error rates" here? The numbers listed here do not appear to match anything else in the paper. Maybe a reference or reminder needs to be included here.

**Authors:** This information is presented under the “Verification abstraction” section at the end of the methods section; we have added additional detail to further clarify what was done and report the outcome of the verification abstraction.

**R1:** Discussion. Page 22: "Actions journals can take to make image-based figures more transparent and easier to interpret". An important item not listed here, but that I personally think is very important, is to add particular requirements about e.g. the use of colorblind-safe colors and inclusion of scale bars in the journal's guidelines for figure preparation/guidelines for authors. Many of these requirements could be listed to the guidelines that many journals already have online. It is much easier to have these requirements up front instead of trying to fix them during the manuscript reviewing stage.

**Authors:** We have updated the discussion to note this, however Existing meta-research suggests that changing journal policies has limited effects on author behavior when there is no plan for implementation or editorial enforcement. Amending journal policies to require authors to report RRIDs, for example, increases the number of papers reporting RRIDs by 1% (Table 2, https://www.ncbi.nlm.nih.gov/pmc/articles/PMC4648211/). In a study of life sciences articles published in Nature journals, the percentage of animal studies reporting the Landis 4 criteria (blinding, randomization, sample size calculation, exclusions) increased from 0 to 16.4% after new guidelines were released (https://openscience.bmj.com/content/3/1/e000035.info). In contrast, a randomized controlled trial of animal studies submitted to PLoS One demonstrated that asking authors to complete the ARRIVE checklist at the time of submission had no effect (https://researchintegrityjournal.biomedcentral.com/articles/10.1186/s41073-019-0069-3). Some improvements in reporting of confidence intervals, sample size justification and inclusion and exclusion criteria were noted after Psychological Science introduced new policies (DOI: 1[0.1371/journal.pone.0175583](https://doi.org/10.1371/journal.pone.0175583)), although this may have been partially due to widespread changes in the field. A joint editorial series published in the Journal of Physiology and British Journal of Pharmacology did not improve the quality of data presentation or statistical reporting (DOI: [10.1371/journal.pone.0202121](https://doi.org/10.1371/journal.pone.0202121)). Editors often note that it is authors’ responsibility to follow the guidelines and reviewers’ responsibility to check; reviewers are overwhelmed and aren’t familiar with the policies of individual journals; authors may or may not carefully examine journal guidelines before submitting. Systemic change is likely going to require automated screening tools that can screen large numbers of manuscripts and flag potential problems. Some of the authors of this paper are working on developing a tool to detect some of the items examined in this paper, but this will take time.

**R1:** Page 23. "of which 500 are estimated to contain images" - do the authors mean photographic images? What is this number based on?

**Authors:** As per the Pubmed statistics site ([https://www.nlm.nih.gov/bsd/index_stats_comp.html)](https://www.nlm.nih.gov/bsd/index_stats_comp.html) and confirmed by a pubmed search, ~ 800,000 new papers are indexed on Pubmed/year, amounting to ~2200/day, 23% of which contain images (Lee et al 2018, REF1). We have replaced the sentence with the following: “Pubmed indexes approximately 800,000 new papers per year, or 2,200 papers per day (<https://www.nlm.nih.gov/bsd/index_stats_comp.html>). Twenty-three percent,^1^ or approximately 500 papers per day, contain images.”

**R1:** Figure 1B and Figure 1C layout could be more similar to each other

**Authors:** We have simplified the layout and legibility of both figures 1 and 2, to have a clearer message, in which process now figures 1B and 1C are more similar.

**R1:** Figure 1C - right hand panel not described in Results, and not clear how it differs from what is shown in the left panel

In Figure 4, Square = 1cm, should this be 1cm2?

Figure 4 refers to 1-3 and 4-6 but there are no numbers in the figure itself.

**Authors:** We have amended the text to add figure 1C to the results and clarify how it differs from what is shown in the left panel and ensure that both panels are clearly explained. In figure 4, we have adapted the text to indicate that the square edge represents 1 cm and added numbers to each panel for clarity.

**R1:** Figure 12: In top right, I did not think the color annotation was that clear ; I liked the solution used in the top left, although that is not color blind safe - could something similar be used in the top right? The line to the mRNA appears to land in an area that has both colors, which was not very clear. Maybe moving it a bit to the left so that it would land in a clear green area would help.

**Authors:** We agree with the reviewer that lines are not ideally suited to indicate colors. We have removed this example.

**R1:** Methods. Page 25, under "Screening" what is meant by "using Rayyan software"? I was not familiar with that tool.

**Authors:** We have amended the description; this is a free, systematic review software that scientists can use to screen articles and note whether they are excluded, along with the reasons for exclusion. It allows for independent assessments by both reviewers, along with automated comparisons to detect discrepancies.

**R1:** Supplemental materials. The Plant Cell articles were included twice in Tables S2 and S3, which was potentially confusing, since now the totals of Tables S1-S3 cannot be summed. I would recommend leaving them out of the Cell Biology table (S3), with a little note under the table, so that there are no duplicate values across the tables.

**Authors:** We considered both options, however we would prefer to include the duplicated journal in both tables so that the total number of included articles match those shown in the graphs.

**We thank the reviewer for pointing out the following minor issues, which have been fixed.**

Page 1, Affiliations of the authors: Typo: "Uterecht"

Introduction. At the end of the Introduction, and the end of "Using a science of science approach...." on Page 4, there are several references to specific figures. I would personally not expect these in the Results, but rather in the Introduction, so maybe consider removing part of that last paragraph of "Using a science...." to the beginning of the Results?

It would be nice if the Abstract should include the total number of papers (580) screened for this study - that number is somewhat hard to find. It is included in Figure S1 (flow chart) and the discussion but it would be good to include it in the abstract and the first paragraph of the Results (see below).

Results. Page 4. It would be more clear to start the Results section by mentioning how many papers (580) were screened.

Figure 4 typo: "Micropcope"

Table S1-S3: maybe include percentages in the top row, e.g., n=409 n=159 (38.9%)

Page 29, under Table S2, should be "This journal was also included on the cell biology list (Table S3)." instead of "(Table S2)".

On page 6, "Approximately half of the papers (47-58%) also failed or partially failed to adequately explain insets. " appears to refer to Figure 1C, right panel, but the figure number/panel is not mentioned. Maybe add that?

Page 11, under 3 "Use Color wisely in images", "Images showing ielectron micrographs" should perhaps read "Images showing electron microphotographs"

Page 13, Maybe write "Deuteranopia, the most common form of colorblindness..." to remind the reader of what the term means (used a lot in the following paragraph)

Discussion. Page  22: "intentionally or accidentally manipulated images" - should be "intentionally or accidentally duplicated images"

Reviewer 2

In general, I find this paper to be excellent and to be potentially a very valuable resource to the community.  I appreciate the large amount of work their initial quantitative findings must have required, and the thoroughness of the recommendations they have put together.

My largest critique (the only one I feel would be NECESSARY to address before publication is that in general), the authors prescribe certain things readers should do when authoring their own papers, but are inconsistent in whether or not they tell readers how to do that (or point them to an educational resource).  This is not universal- they do, for example, point the reader to resources for simulating colorblindness in the text around Figures 7 and 8, but not how to do the inversions or greyscale testing in Figure 6, how to generate labels ala Figures 10 and 11, etc.

Obviously it would be outside the scope of this paper to teach readers to do every task in every POSSIBLE software it could be done in, but the authors could select one or two commonly used tools (such as FIJI, Photoshop/Illustrator, etc, though for maximum utility my vote would be for something free to use) and provide guidance in those.  This could be done along the way, and/or as part of a section at the beginning describing what are some commonly used tools for figure creation (and pointing to resources for each to learn to do common tasks).  In that vein it would also be nice for the authors to more fully credit the tools that were used to make their own figures (they describe which python libraries are used in the creation of their bar graphs, but don't cite the relevant publications for those libraries or for the jupyter project itself (which according to the OSF project is how those figures were created), nor do they describe which software tool(s) they used to create the rest of the figures (They mention the QuickFigures tool at one point, though it's not clear that is what's used in this work or not).

**AUTHORS**: We thank Reviewer 2 for their very relevant suggestion to provide more information regarding tools. While we feel it is indeed far beyond the scope of this article to provide complete details on image processing workflows, we have now included a paragraph in the discussion section detailing possible tools and references for creating legible image figures, and a paragraph in the methods section outlining and referencing the tools we used for image processing in this paper.

New sentence in “Designing image-based figures: How can we improve” section:

“All steps described below (image cropping and insets, adding scale bars and annotation, choosing color channel appearances, figure panel layout) can be implemented with standard image processing software such as FIJI^15^ (RRID:SCR_002285) and ImageJ2^16^(RRID:SCR_003070), which are open source, free programs for bio-image analysis. A quick guide on how to do basic image processing for publications with FIJI is available in a recent cheat sheet publication^17^ and a discussion forum and wiki are available for FIJI and ImageJ (<https://imagej.net/>).”

New sentence in the methods section [after statistical analysis]

Charts were prepared with a Python-based Jupyter Notebook (Jupyter-client, RRID:SCR_018413,^37^ Python version 3.6.9, RRID:SCR_008394, libraries NumPy 1.18.5^38^ and Matplotlib 3.2.2^39^) and assembled into figures with vector graphic software. Example images were previously published or generously donated as indicated in the figure legends. Image acquisition was described in references (*D.melagenoster* images^18^, mouse pancreatic beta islet cells: A. Müller personal communication, and Orobates Pabsti^19^). Images were cropped, labeled, and color-adjusted with FIJI^15^ (RRID:SCR_002285) and assembled with vector-graphic software. Color-blind and grayscale rendering of images was done using Color Oracle^36^ (RRID:SCR_018400). All poor and clear images presented here are ‘mock examples’ prepared based on practices observed during data abstraction.

**R2:**

An additional few smaller critiques-

1) The degree to which the authors obey their own rules for best practices vary; many of the images in the paper lack scale bars, for example, or have illegible bars (figure 6). I understand in most cases that is not the point being illustrated in that particular figure, and would not see it as a blocker for publication, but it would be nice to see them used more consistently, especially in the "good" images.

**Authors:** We thank Reviewer 2 for pointing out inconsistencies between what we preach and what we present and have improved our figures to reflect the standards we uphold. Please see the overall response regarding this topic.

**R2:**

2) The text in the table in Figure 10 is VERY small, it might be better to move it below rather than beside the figure so it can more easily be enlarged.  The text in other figures (such as 9 and 11 is also borderline tiny)

**Authors:** We adjusted the figure layout to place the table below the figure and increase font size.

**R2:**

3) I personally find the broken-up-bar-graph in figure 1B a bit hard to read, especially as the bars for "Some scale bar dimensions" and "All/some magnification in legend" are overlapping; breaking it into multiple bar plots ala 1A lacks the "nice" effect of seeing how things add to 100 but might be more clear.

**Authors:** See also response to reviewer 1. We agree with Reviewer 2 that the legibility could be improved and have simplified the layout and legibility of both figures 1 and 2, to have a clearer message. The details previously shown are now presented in a supplementary table (Table S4).

Reviewer 3

The manuscript starts with quantification of image usage in publications and is followed by quantification of correct/incorrect image reporting (usage of scale information, insets etc.). The analysis of the published papers served the authors to discover problems and to come-up with suggestions that are presented in the following - core part of the manuscript. Here the authors give clear suggestions to relevant steps of image representation and figure preparation. Each step is visualized comparing wrong and right/improved approaches, such that the readers can compare the differences immediately by themselves. The manuscript ends with a final discussion that includes action points suggested to journal and the scientific community. The manuscript is very clearly written and gives the reader clear recommendations on how to improve image display.

**Novelty and significance**

While the single steps addressed (scale bar, color scheme, annotations) are not novel, the way of presenting it with the comparison in figures and the focus on the "colorblind safe" images is. The discussion in context of modern publishing (online) and the connection to online image repositories is timely.

The manuscript gives the reader a very clear "workflow" of what to do in different cases (e.g. 2 color image vs. 3 color image, or EM image vs. color photo) in order to avoid pitfalls. With this I expect it to be of great use, especially (but not only) for early career scientists.

**Points of criticism:**

**R3:** I would have wished for a discussion around the flexibility of the rules and a potential of "miscounting" in the quantification of fig 1. E.g. also in this manuscript the scale bar is missing in most figures and would have been counted accordingly as "Partial scale information" in figure 1. (The reason why the scale bar is missing is written in the text of the manuscript.)

**Authors:** We took several precautions to guard against misclassification errors, as described briefly in the manuscript and in detail in the data abstraction protocol deposited on OSF. First, all abstractors followed the predefined data abstraction protocol. Second, all abstractors completed a training set prior to beginning data abstraction to confirm that results were similar to gold standard ratings. Third, two reviewers abstracted each article. Ratings were compared and disagreements were resolved by consensus, or consultation with TLW if a consensus between abstractors could not be reached. Fourth, verification abstraction was performed by a single abstractor on a random selection of 10% of articles for each field. This confirmed that abstractors in all three fields were using the same procedures throughout the study. Furthermore, the verification abstraction demonstrated that only 3-5% of responses from the consensus decisions of the two abstractors were incorrect.

We have added scale bars to figure 3, 4, 5-8 and 10-13, although the scale bars were de-emphasized to prevent distraction. We have added a sentence in figure 5 explaining why scale bars are not shown, as it is not possible to insert them in a non-distracting way in this figure.

**R3:** Also, I would have wished for a discussion whether/whether not it is important to include details in the figure legend, especially about tissue specification. Under section 7 (prepare figure legends) it is written that some journals require details, while others not - which clearly shows different opinions about this topic. Figure 2B "Are species/tissue/object clearly described in the legend?" shows to me rather different opinions on this topic rather than clear errors in image representation.

**Authors:** We agree that there are different opinions on this topic. We opted for a more stringent criteria for two reasons. First, we needed a criteria that could consistently be applied to all papers in the dataset. Not reporting species in tissue information in the legends of every figure may be less of an issue for papers that examine a single species and tissue, but it is a major problem for studies that include many species and tissues (which may be presented in different panels of the same figure). Second, the scientific community is increasingly developing automated data mining tools (see EMBO’s source data tool) to collect and synthesize information for scientific papers. Unlike humans, these tools do not have the capacity to piece together information scattered throughout the paper to determine what might be shown in a given figure panel. Furthermore, while human readers can do this, it wastes time. We therefore recommend that authors should present information in a clear and accessible manner, even if information is sometimes repeated for studies with simple designs. However, we have no control over journal policies. The discussion reflects the reality that some journals have style guides which directly conflict with our recommendations. We have amended the discussion to explain these nuances.

**Minor comments:**

**R3:** Fig 1: Include to the supplementary examples of images classified as e.g. "insets inaccurately marked, some marked " etc. if this is possible following copyright of already published figures.

**Authors:** Please see the response to all reviewers for information on why we chose to create our own examples based on things seen during abstraction, rather than including actual examples from the dataset.

**R3:** Fig 3A, subcellular scale image is saturated

**Authors:** We replaced this image with a less saturated example of a subcellular scale.

**R3:** Fig 3B. Solution (cell image): inset marking is not fully transparent

**Authors:** Thank you for noting this. We have fixed it.

**R3:** Fig 4: Ruler as scale bar - Square: 1cm; square not visible in this magnification

**Authors:** We replaced the image with a better example.

**R3:** Fig. 5: "Darth Vader being attached" - kids playing Star Wars?

**Authors:** We acknowledge that the legend is a bit hyperbolic for comedic reasons.

**R3:** Section 5. Design the figure: "either from top to bottom and/or from right to left" should presumably read as "left to right"

**Authors:** Thank you for pointing this out - we have fixed it.

**R3:** Fig 6 scale bar not visible in the print as it is for now

**Authors:** We have improved the legibility of the scale bars.

**R3:** Fig 8 Split the color channel: blue described as "least visible" in Fig. 6, but used anyway and

**R3:** Same in Fig. 12 (red), described as "least visible" in Fig. 6, but used anyway

**Authors:** We have started with the ‘typical’ 2- and 3 color images found in the literature and provide options starting from there. We have now included a warning in figure 8 and 12 to indicate that these dark colors on black background are not ideal and that other alternatives are preferable, and referenced Figure 6.

We know from teaching experience that many scientists are uncomfortable presenting certain dyes in alternative colors, e.g. DAPI for staining DNA is typically shown in blue as it emits at ~405 nm, GFP-tagged proteins are shown in green, etc. We therefore wanted to include options that improve the legibility and colorblind safety without changing the default color.

Reviewer 4, Perrine Paul-Gilloteaux

This paper proposes a systematic review of figures in literature in biology-related fields, following some of the PRISMA guidelines, to assess the quality of these published figures. The criteria assessed are the accessibility of figures for color-blindness scientist, the presence of some minimal information as defined by the authors in the legend, the clarity of annotations or insets as assessed by the authors, the presence and clarity of the scale bar. The minimal information (in addition to the scale bar) that should be reported in the legend, as defined by the authors, are defined as the species (or cell lines) observed and the explanation of colors shown. Statistics on the binary fulfilment of these criteria are reported on the selected sample of publications.

The main message reported is that a majority of figures manually inspected by the authors did not fulfil all these criteria.

In addition the authors provides some examples of DO and DON'T for these points and provide guidelines to design good quality figures, according to these criteria.

While the study is certainly a considerable amount of work, and may point out that editors and reviewers did not do their job (PLOS Biology was not assessed)  (reporting scale bar is at least known and required to be present and all figures by editors), I am questioning the choice of the criteria assessed. In particular, authors stated that these criteria serve the reproducibility, I do not understand how badly presented insets may reduce the reproducibility, as stated by the authors. It may unserve the readability, or send a bad message of the rigour of the study, but even this would need to be supported as statements, since in the study the figures which were not filling these criteria did not need them to be understood by the reader. More important guidelines, such as the one asked by journal publishing guidelines (contrast settings, background filtering, process history) would be more important as they can lead to wrong and false messages. The choice of these particular criteria should have been defended against some data or example about how they prevent reproducibility.

**Authors:** The manuscript primarily focuses on legibility, readability and interpretability of image-based figures; reproducibility appears in the keyworks and three times in the discussion. To avoid confusion, we have removed reproducibility from the keywords as well as in two instances in the discussion. The third use has been explained more fully. We agree that there are other factors that are more important for assessing methodological and conceptual reproducibility of image-based data. This study was focused on assessing image-based figures in publications; we did not have access to raw images or meta-data. We agree that the topics raised by the reviewer are important, however they address a different research question and would require a different study design and sampling frame. The QUAREP initiative (<https://quarep.org/>), in which we are participating, is focused on assessing and improving issues related to image methodologies, such as those raised by the authors and plans activities around this topic.

**R4:** Then, showing with the permission of editors/authors, some example of badly assessed figures would have been useful: in particular I am doubtful about the unvisible annotation due to the blending with background color and how it can escape, the example shown of DON'T would serve better the message if taken from real published papers. Real example from real papers of figures assessed as not filling some of the criteria would serve better the message of the paper. Or even more ambitious, adding some reporting on the subjective loss of information and understanding in these papers by the authors of this meta analysis?

**Authors:** While the reviewers understand the desire for “real world” examples, highlighting flaws in published papers written by others is a risky strategy. In addition to potential copyright issues, this also introduces the potential for backlash from authors whose work is highlighted in a negative way (see the threats regularly faced by the first reviewer, Elisabeth Bik who uses real-life examples without even naming authors/citations). We therefore chose to create our own examples, based on problems observed by the data abstractors, to ensure that the paper is solution oriented. We can also use the same images to illustrate poor vs. improved practices, making it easier for authors to compare and contrast. The types of problems shown were selected based on feedback from the data abstractors, who systematically examined every (non-blot) image in hundreds of papers.

Regarding concerns about the types of examples shown, our perception of common practices is often driven by our own expertise, as well as our knowledge of accepted practices for our subfield or the types of images that we work with. Furthermore, the image experts reviewing this paper have important expertise that many scientists who use images lack. Practices that seem obvious to those whose research is centered around images may not be intuitive to those who occasionally use images, but mainly generate other types of data. The advantage of the comprehensive sampling frame used in this study is that we surveyed a wide range of papers that were published in different journals by authors in different fields, subfields, and countries. This provides a more generalizable overview of common practices. The types of problems shown were based on feedback from data abstractors. Scale bars that blend into the background generally occur when authors use dark scale bars on images with dark, mixed color background. Some of this may be driven by scientists using the default scale bar settings, instead of adjusting size, color and labels. Very small red scale bars that are difficult to see, for example, are common because they are the default setting for many Zeiss microscopes.

We agree that data on (subjective) loss of information would be valuable, however this would require a completely different study design from the one that we have used. We would need a carefully curated set of images, in which different versions of the same image contained different types of deficiencies. Study participants would be asked to rate loss of information for various images. Assessing loss of information requires either a very strong baseline image that clearly illustrates all information that the figure is intended to convey, or an intimate knowledge of the field that would allow the reader to assess this information intuitively. Neither of these scenarios were possible in our study, which was designed to assess the prevalence of common problems in a sample selected based on systematic, pre-defined criteria. Attempting to assess information loss without a baseline comparator would be so subjective that the results would be meaningless.

**R4:** For example, even if it is indeed not deserving the main message of the paper, scale bar is not reported in most of the figures of this paper itself (it would have been expected at least for the example of different scale of images Figure 3 ) and in the same time species is reported for all figures when it brings no element to the main message, which is not biologically-related.

**Authors:** We added scale bars to all figures, except figure 3 (a note appears in the legend explaining this). As noted in the manuscript, illustrating every practice described in every figure of the paper would lead to overplotting and make it difficult to identify the key concept shown in any given figure. A central tenet of designing good visualizations is that information that is not essential to the message of the figure should be removed to avoid distraction. This creates some conceptual challenges, including the point recognized by the reviewer (information on species and tissue is not essential to the message of the paper because we are using images to illustrate poor vs. improved practices in general, rather than to show scientific content related to the image itself. We have chosen to model good practice for this particular criteria in every figure, as it affects the legend and does not add clutter to the figure itself).

**R4:** Also in the reporting of the method, I could not get how was defined the error rate mentioned: discrepancy in the binary answer of reviewers on each criteria? Are the scripts to compute the statistics provided? I could not find it on the link provided by the authors.

**Authors:** We have provided additional information in the methods section, in accordance with requests from other reviewers.

**R4:** In addition, one of the main conclusion is also that these recommendations could help in designing the minimal information required when depositing data, but actually the repositories mentioned  (IDR, Cell Atlas) store the raw data, not the figures, so the criteria and factors assessed are not applicable. Could the authors comment on this point or clarify this?

**Authors:** The curated repositories mentioned store the raw images along with microscope acquisition metadata (i.e. scale information, explanation of colors used, staining, species and tissues) and further data concerning the experimental setup, e.g. siRNA sequences used in cell lines, information about the size, the treatment, and organism. Some of the factors assessed in this paper are relevant to the meta-data. Future modifications of repositories may include collecting data from publications. This data, if not provided in the publication, would be missing. We have adjusted this section of the discussion to clarify these points.

**R4:** In conclusion, while the topic is timely relevant in the time of the reproducibility crisis, the authors are sending some messages that should be in the hand of the editors while editing the final proof of papers, in particular with the limited amount and impact of the criteria assessed. The two parts of the paper: constatation of the state of figure published  in April 2018 against the criteria defined by the authors, followed by related guidelines and recommendations, are coherent together but the angle taken is too narrow:, in particular when stating as a main mission the reproducibility of papers. It may be of relevance for teaching courses but I am not sure about its categorization as a research paper as it is. The meta analysis could be of further interest if the support of the message was stronger by proving how this failure in criteria deserves reproducibility and interpretation of the data, as I am not convinced the ones chosen are the more important.

**Authors:** We agree with the reviewer that more extensive assessments examining factors related to image collection, raw image characteristics, image depositing, and loss of information would be helpful. Unfortunately, these combined assessments were beyond the scope of the study, which examined image presentation in published papers and required a lot of time and resources to complete. Research question, sampling frame and study design are intimately linked and the information that the reviewer is requesting would require multiple additional studies, each with a completely different study design and sampling frame from the present study. We have noted the need for research on these additional topics in the limitations and future directions sections of the discussion.

While this study uses a very common meta-research study design, we are aware that these studies seem atypical to the vast majority of scientists, who are not yet familiar with meta-research. Meta-research papers must resonate with scientists outside the meta-research community to have an impact; hence the reviewers’ comments are valuable. It’s important to note that meta-research (the science of science) is different from meta-analysis, in which authors conduct a systematic search to identify all studies addressing a particular research question; then pool the results to estimate the size and direction of the effect based on the literature as a whole.

Reviewer 5, Simon F. Nørrelykke

*** Summary of the research and my overall impression**

** 1. summarise what the ms claims to report

This manuscript details the results from a group of researchers across the globe who got together to document the state of image-based figures in scientific publications. The results obtained show that there is ample room for improvement and the authors proceed by giving figure-creation recommendations that, if followed by authors and journals, should greatly increase the quality of published figures.

Fraudulent image manipulation and how to acquire images is not the focus of this manuscript. Microscopy images, both transmitted, fluorescent, and electron, as well and photographs, are the focus; medical images (MRI, ultrasound, etc) were allowed but rare in the three fields studied.

All papers published during April 2018 in 15 journals (45 journals in total) in the three fields of plant science, cell biology, and physiology were manually examined and scored along several dimensions according to a shared protocol, available online and discussed in the manuscript.

580 papers were examined by "eLife Community Ambassadors from around the world" working together.

Only 2--16% of these papers met all the criteria set for good practices.

Detailed recommendations are given for the preparation of figures with microscopy images. These include discussions of scale bars, insets, colors/colorblindness, label, annotations, legends etc.

Though figures are ideally be designed to reach a wide audience, incl. scientists in other fields, they are typically only interpretable by a very narrow one, if at all.

The advise given on selecting the relevant magnification, how and where to include scale bars, and usage of color, should all be common sense, but apparently is not (behold the results of the investigation reported in this manuscript.) They are thus valuable, even if not novel or thought-provoking, and should be mandatory reading for every student preparing their first manuscript - and perhaps for a majority of PIs, reviewers, and editors alike.

** 2. give overview of the strengths and weaknesses of the ms

- Well written manuscript that reads well (except, perhaps, for the results section)

- The results section is very dry. Six paragraphs lists a large number of percentages. This is data but almost not information. An actuarian may disagree. Figures contain slightly more data and in a more digestible format (graphical).

- Data-acquisition: The number of journals assessed and the approach taken (two reviewers per paper and a clear protocol) is scientific and convincing

- The recommendations are clear and well illustrated

- Though most/all of the points are not new to anyone used to working with images (colorblindness, contrast, scale bars etc), it is useful to see them all collected and commented on in one place - also, every number of years it is useful to remind the community that these things are still (or increasingly? we don't know) an issue.

- Being literal about PLOS criteria:

  + Originality :: this is, as far as I know, the first papers reporting solidly on image-based figure quality

  + Importance to researchers in its field :: Important enough that it should be mandatory reading for any figure-creating scientist

  + Interest to scientists outside the field :: The findings and recommendations cover three fields and easily generalise to other fields

  + Rigorous methodology and substantial evidence for its conclusions :: Yes! Details given elsewhere in report.

**** 3. recommended course of action**

Publish after revision.

Highlight with editorial mention and Twitter activity.

This paper may do more for science than many a pure research manuscript.

* Specific areas that could be improved

**R5: ** Major issues**

- Major, somewhat, because pointing to conceptual issues

  + p. 6 "We evaluated only images in which the authors could have adjusted the image colors (i.e. fluorescence microscopy)"

  + Unless I misunderstand, it is perfectly possible to adjust the colors in any image, so this limitation to fluorescent microscopy images seems to not be justified by the argument given.

  + Example: In an RGB image, e.g. a photo of a flower, the user can set a different color for each of the three channels. This is easily done in, e.g. Imagej/Fiji using the channel tool

* <https://imagej.net/docs/guide/146-28.html#toc-Subsection-28.5>

* [https://imagej.net/docs/guide/146-28.html#sub:Channels...[Z](https://imagej.net/docs/guide/146-28.html#sub:Channels...%5BZ)]

  + Fix: redo research or reformulate sentence to simply state which images you comment on.

  + Or, did you perhaps mean "e.g." and not "i.e."?

**Authors:** We changed “could have adjusted” to “selected”, to clarify that we meant authors had control over the color of the stain used. “i.e.” has been corrected to “e.g.”.

**R5:** Major, but fixable, because pointing to conceptual issues

  + p. 12: "Digital microscope setups capture each channel's intensities in greyscale values."

  + Nope: Some do, some don't.

  + Fluorescent microscopes equipped with filter cubes and very light sensitive CCDs (CMOSs) tend to, as do confocals

  + Slides scanners (also microscopes) are usually equipped with RGB cameras.

  + Suggested fix: delete sentence after understanding why it is wrong

**Authors:** We agree with the reviewer that indeed certain camera’s do capture specific wavelengths/color. We have deleted the misleading sentence from the paper and adjusted the text to note that only some microscopes have this functionality.

**R5:** Suggestion for how to lead by example and in the interest of reproducibility

  + Share the data in an interoperable manner (FAIR principles)

  + Share the Python notebooks used for statistical analysis

  + Share the scripts used to create figures (unless assembled by hand)

  + Do this in GitHub, Zenodo, or the journal website

**Authors:** We have shared code for calculating summary statistics on the OSF repository. As this is an exploratory study, no statistical analyses were performed. Code for the basic panels of figure 1 and figure 2 has been shared. Final labelling and figure assembly was done with vector graphics/ software. Journal websites (supplemental files) are not recommended for data sharing, as many data search engines can’t locate data stored in supplemental files. Materials, including the abstraction protocol, data and code for basic versions of figures 1 and 2, have been shared on OSF, which is a recognized repository backed up by CLOCKSS. Data tags were included on the repository to make the data findable. The repository is currently available to reviewers through a view only link; if the paper is accepted, we will make the repository public and add a DOI to the data citation.

**R5: ** Minor issues**

- p3: EMBO's Source Data tool (RRID:SCR_015018)

  + Is this supposed to be a link or reference?

- p6: "Color Oracle (<https://colororacle.org/>, RRID:SCR_018400)."

  + What is RRID? Not explained until p. 23.

**Authors:** RRIDs, or research resource identifiers, are unique persistent identifiers for software and tools, organisms, plasmids, antibodies, etc. comparable to an accession number of sequence data. These are not abbreviations; they are part of the machine-readable RRID and we have followed standard RRID syntax. Defining an RRID before giving the code would be like defining DOI or ORCID. We welcome the editor’s advice on PLoS Biology’s editorial standards regarding the use of non-standard syntax for papers that report RRIDs.

**R5:** p. 5, Figure 1

  + Please give n in subpanel B, similar to A and C, or Fig 2 A, B, C.

  + Or state that numbers are the same as in A

- p. 11, Figure 4

  + This figure would be more powerful if the problems were 1-1 mirrored by solutions

  + Only two of the five problem images are solved

  + The ruler shown in the bottom right corner is too small to illustrate the point otherwise made: Zooming in, in the pdf, does not give clearly resolved 1cm squares, perhaps due to jpg effect.

  + Alternatively, rename from "problem" and "solution" to something not evoking expectations of solutions to the problems, e.g. by removing those two words.

**Authors:** We have added the sample numbers to figure 1b (previously, the numbers for Fig. 1A and Fig. 1B they were only listed in figure 1A). We have removed “problem” and “solution” to replace and left in place language describing “poor practices” and “better practices”, and replaced the image with the ruler with a better image where the ruler is clearly visible.

**R5:** p. 12, Figure 5, top row

  + This is a very unlikely example of a scientific image

  + Resist temptation of including photos of family members ;-)

  + If you cannot find a natural, scientific, example, perhaps this is not an actual problem?

**Authors:** This figure illustrates the types of images commonly used in scientific papers. As is shown in Figure 1a, photographs of plants, animals, clinical symptoms, experimental set-ups, etc. are very common. We chose a fun example rather than a scientific one; some reviewers enjoyed this, whereas others did not. We welcome the editors input on whether a different image should be selected.

**R5:** p. 12, Figure 5, third and fourth row

  + Recommendations: the splitting should be in addition to, not instead of, adjusting for colorblindness in a merged image

  + Yes, you refer to Fig 8, but here is a natural place to mention it

**Authors:** We agree that scientists should use both splitting and colorblind accessible colors, however there is no splitting in Figure 5. We suspect that the reviewer may have mistaken the two additional columns for channel splitting, when they are actually two different visibility tests (colorblind simulation, grayscale). In row 3, we have used a color palette that is not colorblind safe to illustrate why it’s important to perform visibility tests, including colorblind simulation (shown in column 2). We have added a sentence to the legend to clarify this. Regarding row 4, as noted in figure 8, there are no good color options for colorblind accessibility on a 3-color merged fluorescence image. Figure 5 focuses on when to use color; different image types and scenarios are presented as examples. Once the author has decided that color is needed, she/he can refer to figures 6 to 8 to learn how to use color. Figures 5 and 8 are already quite large. Merging them into a single figure would be very confusing and readers would be missing additional context from figures 6 and 7 that is necessary to understand the concepts illustrated in figure 8.

**R5:** p. 13, Figure 6

  + This figure ought to be redundant, to the extent that the reader knows that higher contrast has higher contrast

  + If, however, the authors saw many examples of dark colors on dark background during their scans of papers, this could still seem a justified figure

**Authors:** We created this figure because a surprising number of authors are unaware of this information. Blue on a black background is surprisingly common (e.g. for DNA- DAPI staining, displayed in blue in default imaging software); red is often used in combination with green on a black background. The highest possible contrast, e.g. inverting colors of a microscopy image to a having white background, is still rarely ever used in figures. We feel this color is a useful resource for authors to encourage them to think about contrast when choosing color schemes.

**R5:** p. 14

  + "Free tools, such as Color Oracle (RRID:SCR_018400)"

  + Also available, for images, in the very popular open source software Fiji under "Image > Color > Simulate Color Blindness"

**Authors:** Thank you for this suggestion; we have added this to the manuscript.

**R5:** p. 15, Figure 8

  + You show possible solutions but do not say what you recommend.

  + Please, do that and argue for the choice!

- p. 16

**Authors:** We amended the figure and figure legend and stated the advantages achieved with each option. We note that the most effective solutions, particularly for 3+ color images, appear at the bottom of the figure. In the paper we are not arguing for one particular option as the gold standards as other factors (journal guidelines, standard practice in lab, space restrictions) also influence the ultimate decision.

**R5:** + "QuickFigures (RRID:SCR019082)"

  + Does this software support reproducibility (creates scripts that can generate entire figure)?

  + Please comment in manuscript

**Authors:** We removed this sentence from the revised manuscript.

**R5:** p. 17, Figure 10

  + Text in right half of figure is too small to comfortably read

**Authors:** We restructured the figure so that the images appear on top of the table and the text in the table is larger.

**R5:** p. 21 Figure 13

  + Add title to third column

**Authors:** We added a title to this column and adjusted the space between columns for clearer delineation.

**R5:** p. 23 "increase training opportunities in bioimaging" should, likely, read "increase training opportunities in bioimage analysis"

**Authors:** We adjusted the language.

**R5:** p. 35, Figure S1 - Please create higher quality figure that better supports zooming in

Authors: We have created a higher quality version of this figure.

**R5:** Suggestion - Cite first author's recent paper in F1000R-NEUBIAS on same topic

**Authors:** We have included the requested reference.
